# Evaluating the efficacy and safety of an all-inside, all-suture meniscal repair device on arthroscopic meniscal repair: a prospective, multi-center, randomized controlled clinical trial

**DOI:** 10.1097/JS9.0000000000004608

**Published:** 2026-02-11

**Authors:** Lin-Yi Shen, Yunshen Ge, Ziyin Wu, Shurong Zhang, Fang Wan, Xiaofei Zheng, Huige Hou, JieRuo Li, Jing Wang, Yuanjie Zeng, Zhen Jia, Zhizeng Gao, Wenzhao Chen, Fengjin Guo, Hongyun Li, Shiyi Chen

**Affiliations:** a Department of Sports Medicine, Huashan Hospital, Shanghai, China; b Department of Joint Surgery and Sports Medicine, The First Clinical Medical College of Jinan University, The First Affiliated Hospital of Jinan University, Guangzhou, China; c Department of Joint and Sports Medicine, Hunan Provincial People’s Hospital, Changsha, China; d Department of Sports Medicine, The First Affiliated Hospital of Nanchang University, Nanchang, China; e Department of Joint Surgery, Union Hospital, Tongji Medical College, Huazhong University of Science and Technology, Wuhan, China

**Keywords:** all-inside, all-suture, arthroscopic meniscal repair, randomized controlled clinical trial

## Abstract

**Background:**

To evaluate the efficacy and safety of an all-inside, all-suture meniscal repair device for arthroscopic meniscal repair (AMR).

**Materials and methods:**

Patients with vertical longitudinal full-thickness meniscus tears undergoing AMR were randomly assigned to the intervention group (all-inside, all-suture meniscal repair device) or the control group (all-inside meniscal repair system). The primary efficacy endpoint was the Lysholm Knee Score (LKS) at 6 months postoperatively. Secondary efficacy endpoints included: (1) the immediate device success rate at intraoperatively; (2) LKS at 3 and 12 months postoperatively; (3) Tegner activity score (TAS) and visual analogue scale (VAS) of pain at 3, 6, and 12 months postoperatively; (5) Meniscus repair status evaluated by magnetic resonance imaging (MRI) at 6 and 12 months postoperatively. Adverse events (AEs) and device deficiencies were recorded to assess safety. Final follow-up for 12 months was completed in April 2024.

**Results:**

A total of 94 patients from 5 tier-A centers in China were randomized (intervention group: *n* = 48; control group: *n* = 46). Primary outcome analysis included 91 patients (intervention group: *n* = 45; control group: *n* = 46). LKS improved significantly from baseline in both groups at 6 months (both *P* < 0.001), with no between-group difference in absolute scores (mean ± standard deviation: 90.85 ± 8.70 vs 90.96 ± 11.52, *P* = 0.503). However, covariance analysis revealed greater LKS improvements in the intervention group (mean ± standard deviation: 30.17 ± 1.65 vs 23.87 ± 1.93, *P* = 0.015). Safety analysis showed no significant difference in the incidence of surgery-related AEs between the groups (17.02% vs 17.39%, *P* = 1.000).

**Conclusion:**

The all-inside, all-suture meniscal repair device demonstrated comparable efficacy and safety to the controlled all-inside meniscal repair device for arthroscopic repair of vertical longitudinal full-thickness meniscus tears over 12 months. Moreover, it showed superior early improvement in knee function recovery and can be considered an alternative for AMR.

## Introduction

The meniscus is a crescent-shaped fibrocartilaginous structure in the knee joint that increases the depth of the articular surfaces, compensates for mismatches between articular surfaces, and provides important meniscal biological functions through ring-parallel peripherals^[1]^. As one of the most commonly injured sites in the knee, meniscus preservation is essential for maintaining knee function^[2]^.

Meniscal injuries, caused by trauma, degeneration or congenital anomalies, are common across various age groups and activity levels. They can cause clinical symptoms such as pain, knee locking, limited mobility, as well as physical damage^[3–5]^. Patients with persistent mechanical symptoms and/or pain unresponsive to conservative treatment should consider surgical intervention^[6]^. Before 1960, open meniscectomy was the primary approach to manage meniscal tears^[7]^. Currently, surgical options include debridement, meniscectomy, repair and reconstruction^[8]^.

With the rapid advancement of surgical techniques and instruments, AMR has become the preferred treatment option for meniscal tears^[9,10]^. The success of meniscus repair depends on injury type, injury size, location, blood supply, repair technique and concomitant lesions.^[11–13]^ Nowadays, meniscus repair can be performed using three primary arthroscopic techniques: inside-out, outside-in or all-inside repair^[8,11,14,15]^. With increasing awareness of meniscus preservation, various devices have been developed to facilitate the all-inside technique, which avoids additional incisions, reduces neurovascular injury risks and shortens operating times^[14,16,17]^.

Traditional meniscal knotting and fixation devices often use rigid plastic anchors and surface junctions, which may cause complications such as cartilage damage, synovitis, as well as anchor displacement, fragmentation, and soft tissue stimulation^[7]^. To avoid these complications associated with rigid plastic anchors and hard meniscal surface knots, and to allow for controlled tension, an all-inside, all-suture meniscal repair device was designed. This device features two soft polyester suture anchors linked by a 2–0 ultra-high molecular weight polyethylene (UHMWPE) adjustable locking suture, deployed via a 1.6-mm needle inserter. The anchors expand post-deployment on the extra-articular side of the knee capsule, providing biomechanical advantages such as higher failure loads and reduced elongation compared to traditional rigid PEEK-anchored or inside-out suture methods, as demonstrated in cadaveric and porcine studies^[17,18]^. The knotless design minimizes suture cutout risk, ensures uniform tension distribution, and simplifies the procedure by eliminating capsular knot-tying, potentially lowering complications like cartilage irritation or synovitis.

Our study aimed to investigate the clinical efficacy and safety of the all-inside, all-suture meniscal repair device compared to the all-inside meniscal repair system. The controlled repair system has already been demonstrated high acceptance in meniscus repair^[19–21]^. We hypothesized that the all-inside all-suture meniscal repair system would have comparable outcomes.

This RCT is in compliance with the TITAN Guidelines 2025 for transparency in the reporting of artificial intelligence^[22]^.

## Material and methods

### Study design

This prospective, multicenter, randomized controlled clinical trial (RCT) evaluated the efficacy and safety of the all-inside, all-suture meniscal repair device in patients with vertical longitudinal full-thickness meniscus tears. The trial protocol was approved by the ethics committee of the 5 participating centers (Supplemental Digital Content eTable 1 in Supplement 1, available at: http://links.lww.com/JS9/G886). All participants provided written informed consent. This clinical trial followed the Consolidated Standards of Reporting Trials (CONSORT) reporting guidelines^[23]^.

### Participants

A total of 94 patients were enrolled from 5 class A tertiary hospitals in China from February 2022 to April 2023. All the inclusion and exclusion criteria are detailed in Supplemental Digital Content eTable 2 in Supplement 1, available at: http://links.lww.com/JS9/G886. For participants to be screened successfully, diagnostic arthroscopy would be scheduled to confirm the eligibility intraoperatively prior to randomization.

**HIGHLIGHTS**

- A multicenter RCT compared an all-inside, all-suture meniscal repair device with a control system in 94 patients with vertical longitudinal meniscal tears.
- At 6 months, Lysholm scores improved significantly in both groups, with greater early functional recovery in the all-inside, all-suture meniscal repair device group (adjusted *P* = 0.015).
- Safety profiles were comparable in two groups, with no significant differences in adverse event rates.
- The device showed non-inferior efficacy and safety over 12 months, suggesting it as a viable alternative for arthroscopic meniscal repair.

## Diagnostic arthroscopy, randomization and meniscus repair

The surgery was performed according to the standard arthroscopic procedure, with medial and lateral approaches to the knee joint. Under general or lumbar anesthesia, patients were placed in the supine position upon surgeon’s preference, and a routine tourniquet was placed to the upper and middle thigh. A gauge spinal needle was used under direct arthroscopic visualization to optimize portal placement, ensuring the portal entered just above the meniscus parallel to the tibial joint surface. Portals were positioned to avoid being too superior or inferior, and the medial portal was sufficiently larger to accommodate device insertion without soft tissue interference. For mid-body tears of the medial meniscus, the scope was switched to the medial portal, and the lateral portal was assessed and expanded or recreated if necessary to ensure parallel access just above the anterior lateral meniscus. A comprehensive examination of the knee joint was conducted to precise locate the meniscus tear, confirm its vertical longitudinal full-thickness morphology in the red–red or red–white zone, and evaluate reparability.

Eligible patients were randomized intraoperatively to the intervention group (JuggerStitch^®^ meniscal repair device, Zimmer Biomet, Warsaw, IN) or control group (Fast-Fix 360 meniscal repair system, Smith & Nephew Endoscopy, Andover, MA) utilizing a central randomization system with stratification for concomitant anterior cruciate ligament (ACL) injury, using a computer-generated sequence to balance groups. When handling a meniscus injury, any loose body within the joint were removed, and the joint was cleaned. A probe was used to select the appropriate suture device based on the location of the meniscus injury.

The repair technique was standardized across centers and surgeons, who underwent protocol-specific training. Suture spacing was maintained at 3–5 mm between points to optimize tear apposition, even tension distribution, and biomechanical stability, preventing gap formation while aligning with clinical guidelines for all-inside repairs. No aiming guides were used, as device designs and arthroscopic visualization ensured precision. The suture configuration was a vertical mattress pattern, engaging circumferential fibers for enhanced load-to-failure strength in longitudinal tears. Suture passage was uniformly from the intra-articular side (starting from either femoral or tibial surface) to the extra-articular capsular side, without direct femoral-to-tibial traversal within the meniscus. Needle entry was initiated 3–5 mm from the tear edge, with the first puncture on the inferior (tibial) rim below the tear and the second on the superior (femoral) rim above it. The intraoperative puncture pathway was perpendicular to the meniscus surface to ensure even anchor deployment and tension, confirmed by a “breaking through” sensation upon capsular penetration. For the intervention group, the JuggerStitch device’s adjustable depth slider was set between 10 and 18 mm (default 18 mm, adjusted based on meniscal thickness at the tear site) to ensure full needle penetration to the capsule without excessive depth risking neurovascular structures. The 1.6-mm needle inserter deployed two soft polyester anchors linked by a 2–0 ultra-high molecular weight polyethylene (UHMWPE) adjustable locking suture. The first anchor was deployed extracapsularly via needle penetration from the tibial surface, followed by suture tensioning to reduce the tear. The second anchor was then deployed from the femoral surface, with final knotless tensioning to compress the repair uniformly, minimizing suture cutout and ensuring even distribution without external incisions or knot-tying. For the control group, the Fast-Fix 360 system deployed rigid PEEK anchors connected by a pre-tied sliding knot suture. The curved or straight needle inserter penetrated the meniscus perpendicularly, deploying the first anchor extracapsularly from the tibial surface. The device was repositioned for the second anchor from the femoral surface, followed by pulling the suture tail to advance the sliding knot and tension the repair, achieving compression and stability. For all patients in the two groups, a half-pipe cannula facilitated device insertion through the portal, and a probe or hook aided tear reduction, repositioning, and fixation during tensioning. Multiple stitches (typically ≥2) were placed based on tear size to achieve stable. After repair, the meniscus was probed to confirm that the meniscus was securely fixed, the soft anchor and suture were stable, and the incision was flushed and closed. ACL reconstruction was performed if ACL rupture was identified during the arthroscopic evaluation. The reconstruction was carried out using either a four-strand autogenous hamstring tendon graft or a Ligament Augmentation Reinforcement System (LARS) artificial ligament, depending on the surgeon’s preference and the patient’s specific condition.

## Follow-up schedule and rehabilitation protocol

Patients in both groups followed a standardized follow-up and rehabilitation protocol.

The postoperative rehabilitation for meniscal suture surgery was recommended to all patients as follows:

- *Within 3 weeks*: Patients performed ankle pumps, tightened their thigh muscles with the knee straightened, and raised the straight leg to 30–50 degrees.
- *Weeks 4–8*: Patients started riding a stationary bike, performed straight leg elevations with added resistance, tightened their leg muscles at different angles, did elastic belt exercises, stepped up and down, and performed wall-supported half-squats.
- *After 2 months*: Patients used a training device for joint resistance, underwent gait training, and participated in hydrotherapy.

Range of motion exercises was as follows:

- *Within 0–4 weeks*: Knee flexion was limited to less than 90 degrees.
- *Within 4–6 weeks*: Knee flexion increased to less than 120 degrees.
- *After 6 weeks*: Patients returned to normal knee flexion.

Patients should avoid squats for 3 months postoperatively and sudden knee movements for 6 months. Weight-bearing started with crutches and partial weight-bearing, progressing to full weight-bearing over 6 weeks. Cold compresses were applied every 2–3 hours in the first week, then reduced based on swelling. Patients rested for 10–15 minutes after each exercise session.

Phone call visit at 3 months and outpatient visits at 6 and 12 months were scheduled to assess the LKS, TAS and VAS. Assessments on these PROMs were unblinding since they required interaction with the patient and access to relevant clinical history for context and safety monitoring.

MRI was performed at 6 and 12 months to evaluate the meniscus healing status. This study established an independent MRI imaging central laboratory to guide all centers in collecting and transmitting MRI data and to independently evaluate MRI results from the perspective of quality control. The evaluators in the central laboratory were blinded to the group allocation. Meniscal healing was assessed using the Stoller grading system on MRI: Grade 0 (normal meniscus), Grade I (focal globular or elliptical hyperintense signal not reaching the articular surface), Grade II (horizontal linear hyperintense signal extending to the microcapsular junction), and Grade III (linear hyperintense signal reaching the articular surface, indicating retear)^[24]^.

## Clinical outcomes

### Efficacy outcomes

The primary efficacy outcome was the improvement in LKS from baseline to 6 months postoperatively. The secondary efficacy outcomes included: (1) the immediate device success rate (Immediate device success was uniformly defined across centers as successful intraoperative deployment and stable fixation of the meniscus upon probing, with no immediate redeployment required due to anchor failure, suture slippage, or instability); (2) LKS at 3 and 12 months postoperatively; (3) TAS and VAS at 3, 6, and 12 months postoperatively; (4) meniscus repair status evaluated by MRI at 6 and 12 months postoperatively.

### Safety outcomes

Safety outcomes included all adverse events, surgery-related adverse events, and device-related adverse events occurred during surgery and follow-up period.

### Sample size and statistical analysis

The sample size was calculated based on a noninferiority design for the primary endpoint of the change in LKS from baseline to postoperative 6 months. Based on prior studies, the expected change was 30 points,^[25–30]^ but conservatively assumed as 34 for both groups, with a standard deviation (SD) of 8. Using a one-sided test level (α) of 0.025, statistical power (β) of 0.8, and a noninferiority margin (δ) of 5.1 (15% of the assumed difference), the total sample size was determined to be 40 cases per group, with an additional 15% to account for drop out, resulting in 47 cases per group. Sample size was not adjusted for design effects from stratification, as randomization was at the individual level and aimed to balance groups rather than account for clustering.

Quantitative variables were described using mean and SD. Qualitative variables were described by calculating the number of cases and percentages in each category.

The Full Analysis Set (FAS) included all subjects who provided informed consent and underwent surgery with randomized meniscal repair system. Secondary endpoints were analyzed based on the actual observed data without imputation. For the primary endpoint, the modified mean (LS means) and 95% confidence interval (CI) of the difference in LKS at 6 months between the groups were estimated using an analysis of covariance (ANCOVA) model with baseline LKS and age included as covariates. For subjects who did not complete the LKS at postoperative 6 months, the last observation carried forward (LOCF) method was used to estimate the outcome. To assess the robustness of the results, a complete-case Per-Protocol Set (PPS) analysis was also performed, excluding the missing data point.

Baseline characteristics were analyzed descriptively using the FAS, and group comparisons were made using t-tests, Wilcoxon tests, and *χ*^2^ tests. Efficacy indicators were analyzed using both the PPS and FAS analysis sets.

Safety assessments were based on the Safety Set (SS) analysis set. AEs were documented and analyzed for correlation with procedures and products. Statistical analysis was performed using SAS 9.40 software. A *P*-value threshold of less than 0.05 was established to define statistical significance.

## Results

### Distribution of patients and baseline characteristics

**Figure 1.**
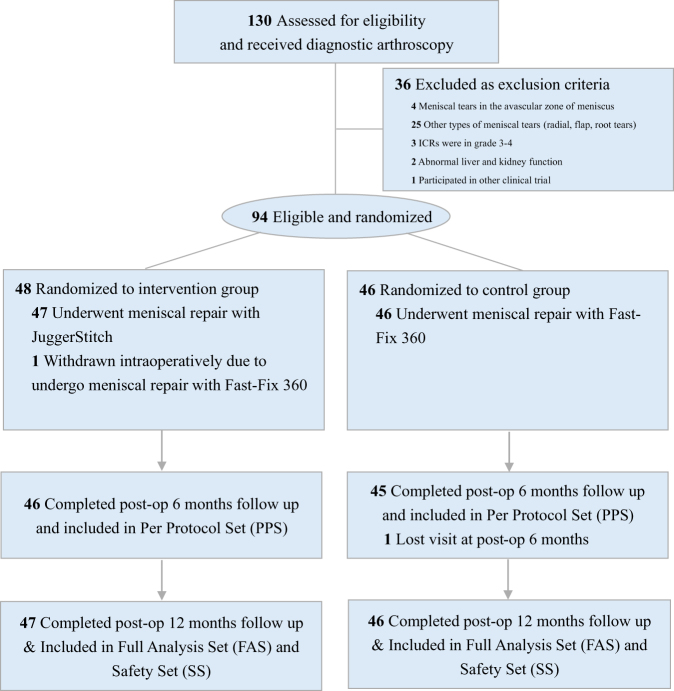
CONSORT flow diagram.

A total of 130 patients were screened and received diagnostic arthroscopy to confirm eligibility per the study protocol. Ultimately, 94 patients were qualified and randomized intraoperatively (intervention group = 48; control group = 46). Of these, 93 patients underwent arthroscopic repair using randomized devices and were included in the FAS ad SS analysis (intervention group = 47; control group = 46). Additionally, 91 patients (intervention group = 46; control group = 45) achieved the primary outcome and were included in the PPS analysis (Fig. 1).

ICRs, International Cartilage Repair Society.

Based on FAS analysis, 93 patients included a higher proportion of male (female: 29.03% vs male: 70.97%) with a mean (SD) age of 32.47 (9.24) years. There was no significant difference between the groups in the distribution of gender, weight, height or BMI (all *P* > 0.05). However, age differed significantly between the groups (*P* = 0.034; Table 1). The analysis also revealed no significant differences between the groups in physical examinations and radiographic findings at baseline (all *P* > 0.05; Table 1).

**Table 1:** Patient demographics and clinical characteristics.

Parameter	Intervention group	Control group	*P-*Value
*Patient demographics*	*n* = 47	*n* = 46	
Age (years), mean (SD)	30.47 (8.63)	34.52 (9.48)	0.034*
Gender, No. (%)			
Male	31 (65.96%)	35 (76.09%)	0.282
Female	16 (34.04%)	11 (23.91%)	
BMI (Kg/m^2^), mean (SD)	23.53 (3.12)	24.81 (3.65)	0.117
*Baseline physical examinations*			
Joint motion, No. (%)	*n* = 47	*n* = 46	
Positive	46 (97.87%)	46 (100.00%)	1.000
Negative	1 (2.13%)	0 (0.00%)	
Positive extension (°), mean (SD)	1.20 (3.68)	0.98 (2.27)	0.652
Negative extension (°), mean (SD)	5.00 (.)	-	-
Joint motion-flexion (°), mean (SD)	124.04 (18.99)	126.09 (17.09)	0.252
Pain in interarticular pressure, No. (%)	*n* = 47	*n* = 44	
Positive	9 (19.15%)	7 (15.91%)	0.685
Negative	38 (80.85%)	37 (84.09%)	
Swelling, No. (%)	*n* = 47	*n* = 46	
Positive	31 (65.96%)	33 (71.74%)	0.547
Negative	16 (34.04%)	13 (28.26%)	
Lever sign, No. (%)	*n* = 45	*n* = 43	
Positive	19 (42.22%)	16 (37.21%)	0.631
Negative	26 (57.78%)	27 (62.79%)	
Anterior drawer test, No. (%)	*n* = 47	*n* = 44	
Positive	11 (23.40%)	11 (23.91%)	0.558
Negative	36 (76.60%)	33 (71.74%)	
Lachamn test, No. (%)	*n* = 46	*n* = 46	0.732
Positive	12 (25.53%)	14 (30.43%)	
Negative	34 (72.34%)	32 (69.57%)	
Valgus stress test, No. (%)	*n* = 47	*n* = 46	1.000
Positive	46 (97.87%)	45 (97.83%)	
Negative	1 (2.13%)	1 (2.17%)	
Varus stress test, No. (%)	*n* = 47	*n* = 46	
Positive	47 (100.00%)	46 (100.00%)	-
Negative	0 (0.00%)	0 (0.00%)	
*Baseline radiographic findings*	*n* = 47	*n* = 46	
Grading of arthritis, No. (%)			
Class 0	47 (100.00%)	44 (95.65%)	0.242
Class I	0 (0.00%)	1 (2.17%)	
Class II	0 (0.00%)	1 (2.17%)	
Class III	0 (0.00%)	0 (0.00%)	
Class IV	0 (0.00%)	0 (0.00%)	
Abnormal in medial meniscus, No. (%)			
No	14 (29.79%)	10 (21.74%)	0.375
Yes	33 (70.21%)	36 (78.26%)	
Abnormal in lateral meniscus, No. (%)			
No	19 (40.43%)	15 (32.61%)	0.434
Yes	28 (59.57%)	31 (67.39%)	
ACL injury, No. (%)			
No	7 (14.89%)	5 (10.87%)	0.563
Yes	40 (85.11%)	41 (89.13%)	

BMI, body mass index; SD, standard deviation.

* *P* < 0.05

### Surgical outcomes

In the intervention group, 24 (48.94%) left knees and 23 (51.06%) right knees underwent arthroscopic meniscus repair, compared with 23 (50.00%) left knees and 23 (50.00%) right knees in the control group. For patients with medial or lateral meniscus tears, there were no statistically significant differences in the percentage of tears, tear zoning or tear type between the groups (all *P* > 0.05). However, the length of the lateral meniscus tear was statistically significant between the groups (*P* = 0.038). In addition, the difference in the proportion and degree of cartilage damage and concomitant ACL injuries did not present significant difference (all *P* > 0.05). The average number (SD) of implanted devices were 3.45 (1.63) in the intervention group and 3.11 (1.57) in the control group, indicating no significant difference between the groups (*P* = 0.330; Table 2).

**Table 2:** Surgical outcomes.

Parameter	Intervention group	Control group	*P*-Value
Affected side, No. (%)	*n* = 47	*n* = 46	
Left knee	23 (48.94%)	23 (50.00%)	0.918
Right knee	24 (51.06%)	23 (50.00%)	
Medial meniscus tear, No. (%)	*n* = 47	*n* = 46	
No	10 (21.28%)	11 (23.91%)	0.761
Yes	37 (78.72%)	35 (76.09%)	
Length of medial tear (mm), mean (SD)	14.97 (7.12)	16.06 (7.01)	0.513
Medial tear level, No. (%)	*n* = 37	*n* = 34	
Partial tear	0 (0.00%)	0 (0.00%)	-
Full tear	37 (100.00%)	34 (100.00%)	
Medial tear zone, No. (%)	*n* = 37	*n* = 34	
Red–red zone	33 (89.19%)	31 (91.18%)	1.000
Red–white zone	4 (10.81%)	3 (8.82%)	
White-white zone	0 (0.00%)	0 (0.00%)	
Lateral meniscus tear, No. (%)	*n* = 47	*n* = 46	
No	21 (44.68%)	17 (36.96%)	0.449
Yes	26 (55.32%)	29 (63.04%)	
Length of Lateral tear (mm), mean (SD)	17.00 (8.38)	14.46 (8.25)	0.224
Lateral tear level, No. (%)	*n* = 25	*n* = 27	
Partial tear	0 (0.00%)	1 (3.70%)	1.000
Full tear	25 (100.00%)	26 (96.30%)	
Lateral tear zone, No. (%)	*n* = 25	*n* = 26	
Red–red zone	17 (68.00%)	24 (92.31%)	0.038
Red–white zone	8 (32.00%)	2 (7.69%)	
White-white zone	0 (0.00%)	0 (0.00%)	
Cartilage damage, No. (%)	*n* = 47	*n* = 46	
No	15 (31.91%)	16 (34.78%)	0.769
Yes	32 (68.09%)	30 (65.22%)	
Cartilage damage degree, No. (%)	*n* = 32	*n* = 30	
Degree I	5 (15.63%)	6 (20.00%)	0.652
Degree II	27 (84.38%)	24 (80.00%)	
ACL injury, No. (%)	*n* = 47	*n* = 46	
No	9 (19.15%)	11 (23.91%)	0.348
Yes	38 (80.85%)	35 (76.09%)	
Number of implanted devices, No. (%)	*n* = 47	*n* = 46	
1 anchor	6 (12.77%)	9 (19.57%)	0.668
2 anchors	10 (21.28%)	8 (17.39%)	
3 anchors	9 (19.15%)	13 (28.26%)	
4 anchors	7 (14.89%)	4 (8.70%)	
5 anchors	9 (19.15%)	9 (19.57%)	
6 anchors	6 (12.77%)	3 (6.52%)	
Mean (SD)	3.45 (1.63)	3.11 (1.57)	0.330
Location of implanted lesion, No. (%)			
Medial meniscus	102 (62.58%)	90 (63.83%)	0.821
Lateral meniscus	61 (37.42%)	51 (36.17%)	
Success rate, (%)^a^	84.15%	91.49%	0.053
User evaluation, No. (%)	*n* = 47	*n* = 46	
Satisfaction	38 (80.85%)	38 (82.61%)	0.826
More satisfactory	9 (19.15%)	8 (17.39%)	
General satisfaction	0 (0.00%)	0 (0.00%)	
Dissatisfied	0 (0.00%)	0 (0.00%)	

ACL, anterior cruciate ligament; SD, standard deviation.

^a^ Immediate device success rate assessed intraoperatively as complete anchor deployment and fixation without slippage, pull-out, or locking issues

### Primary efficacy outcome

The baseline LKS was included as a covariate in the analysis. Based on PPS analysis, the mean (SD) LKS scores at 6 months postoperatively were 90.85 (8.70) in the intervention group and 90.96 (11.52) in the control group, with no statistically significant difference between the groups (*P* = 0.670). The mean (SD) change scores of total LKS were 30.17 (1.65) in the intervention group and 23.87 (1.93) in the control group, with a mean difference of 6.30 (95% CI: 1.25–11.34, *P* = 0.015), which showed superior knee function improvement in the intervention group compared with the control group. A complete-case sensitivity analysis confirmed these findings (mean difference: 6.30, 95% CI: 1.25–11.34, *P* = 0.015). Moreover, the results demonstrated that LKS at 6 months postoperatively had significantly improved in both groups, with a highly significant difference compared with preoperative scores (both *P* < 0.001; Table 3). Including age as a covariate in the ANCOVA model yielded similar results (adjusted mean difference: 6.25, 95% CI: 1.15–11.35, *P* = 0.016).

**Table 3:** Lysholm Knee Score (LKS, 0–100) at baseline and postoperative 6 months for the two groups.

	FAS			PPS		
Outcome mean (SD)	Intervention group (*n* = 47)	Control group (*n* = 46)	*P*-Value	Intervention group (*n* = 46)	Control group (*n* = 45)	*P*-value
Baseline	56.57 (14.67)	57.70 (14.28)	0.710	56.57 (14.67)	57.70 (14.28)	0.710
6 months	89.94 (10.64)	90.11 (12.75)	0.517	90.85 (8.70)	90.96 (11.52)	0.503
Improved scores (baseline to 6 months)	33.36 (17.06)	32.41 (20.42)	0.808	34.13 (16.40)	33.78 (18.40)	0.923
*P*-value	<0.001*	<0.001*		<0.001*	<0.001*	
Adjusted improved scores (baseline to 6 months)^a^	29.51 ± 2.10	23.17 ± 2.47	0.053	30.17 ± 1.65	23.87 ± 1.93	0.015*
Group difference (95% CI)	6.34 (−0.08–12.76)			6.30 95% CI: 1.25–11.34		

FAS, full analysis set; PPS, per protocol set; SD, standard deviation.

* *P* < 0.05

^a^ The baseline LKS was included as a covariate in the analysis and conducted covariance analysis to adjust improved scores for the groups.

To assess clinical significance, the proportion of patients achieving the MCID (≥10-point improvement in LKS from baseline) was calculated^[31]^. Under the PPS analysis, 95.7% (45/47) in the intervention group and 89.1% (41/46) in the control group at 6 months achieved MCID (*P* = 0.431; Table 4).

**Table 4:** The proportion of patients achieving MCID in each group.

Intervals	FAS			PPS		
	Intervention group (% achieved)	Control group (% achieved)	*P*-Value	Intervention group (% achieved)	Control group (% achieved)	*P*-Value
3 months	78.72% (37/47)	76.09% (35/46)	0.478	78.26% (36/46)	77.78% (35/45)	0.578
6 months	91.49% (43/47)	86.96% (40/46)	0.356	91.30% (42/46)	86.67% (39/45)	0.356
12 months	93.62% (44/47)	93.18% (41/44)	0.629	93.48% (43/46)	93.18% (41/44)	0.640

FAS, full analysis set; PPS, per protocol set; SD, standard deviation

### Secondary efficacy outcomes

#### Immediate implant success rate

Based on the FAS analysis, the immediate implant success rate was 84.15% (138/164) in the intervention group and 91.49% (129/141) in the control group, with no statistically significant difference between the groups (*P* = 0.053; Table 2). Regarding the evaluation of the intraoperative implant performance, the satisfaction rate was 100% for both groups, that reflected positive device implantation and user experience with both meniscal repair systems.

#### Patient reported outcomes

**Figure 2.**
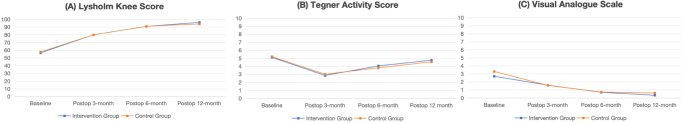
The line chart presented longitudinal trends in PROs.

No statistically significant differences were found in the total LKS at 3 months and 12 months, TAS and VAS at 3, 6, and 12 months under either FAS or PPS analysis (all *P* > 0.05). All PROMs of both groups improved significantly at 12 months postoperatively were observed in the changes between the groups (all *P* < 0.001), but no significant differences were found in the changes between the groups (all *P* > 0.05; Table 5). The line chart presented longitudinal trends in PROs (Fig. 2).

**Table 5:** Clinical outcomes on secondary endpoints for the two groups.

		FAS			PPS		
Outcomes mean (SD)	Interval	Intervention group (*n* = 47)	Control group (*n* = 46)	*P*-Value (groups)	Intervention group (*n* = 46)	Control group (*n* = 45)	*P*-Value (groups)
LKS	3 months	79.51 (13.76)	79.26 (13.73)	0.930	79.89 (13.66)	79.87 (13.25)	0.993
	Improvement from baseline	22.94 (20.07)	21.57 (20.42)	0.745	23.17 (20.22)	22.69 (19.15)	0.907
	*P*-value (pre–post)	< 0.001*	< 0.001*		< 0.001*	< 0.001*	
	12 months	95.89 (4.83)	94.02 (8.11)	0.349	96.02 (4.80)	94.02 (8.11)	0.291
	Improvement from baseline	39.65 (15.00)	36.64 (15.93)	0.925	39.64 (15.17)	36.64 (15.93)	0.912
	*P*-value	< 0.001*	< 0.001*	0.413	< 0.001*	< 0.001*	
TAS	Baseline	5.11 (1.31)	5.20 (1.51)	0.997	5.11 (1.31)	5.20 (1.51)	0.997
	3 months	2.85 (0.93)	3.02 (0.93)	0.371	2.85 (0.94)	3.00 (0.93)	0.437
	Improvement from baseline	−2.26 (1.34)	−2.17 (1.54)	0.606	−2.24 (1.35)	−2.18 (1.56)	0.678
	*P*-value	<0.001*	<0.001*		<0.001*	<0.001*	
	6 months	4.02 (1.19)	3.80 (1.08)	0.570	4.04 (1.19)	3.80 (1.08)	0.495
	Improvement from baseline	−1.09 (1.53)	−1.38 (1.57)	0.420	−1.04 (1.52)	−1.38 (1.57)	0.342
	*P*-value	<0.001*	<0.001*		<0.001*	<0.001*	
	12 months	4.74 (1.47)	4.55 (1.44)	0.511	4.76 (1.48)	4.55 (1.44)	0.483
	Improvement from baseline	−0.41 (1.65)	−0.64 (1.83)	0.662	−0.38 (1.66)	−0.64 (1.83)	0.583
	*P*-value	0.071	0.026*		0.099	0.026*	
VAS	Baseline	2.70 (1.61)	3.30 (1.72)	0.060	2.70 (1.61)	3.30 (1.72)	0.074
	3 months	1.60 (0.95)	1.59 (1.09)	0.785	1.59 (0.96)	1.56 (1.08)	0.699
	Improvement from baseline	−1.11 (1.67)	−1.72 (1.91)	0.084	−1.13 (1.68)	−1.76 (1.91)	0.079
	*P*-value	< 0.001*	< 0.001*		< 0.001*	< 0.001*	
	6 months	0.72 (0.71)	0.73 (0.65)	0.138	0.70 (0.70)	0.73 (0.65)	0.322
	Improvement from baseline	−1.98 (1.59)	−2.58 (1.79)	0.097	−2.02 (1.58)	−2.58 (1.79)	0.129
	*P*-value	< 0.001*	< 0.001*		< 0.001*	< 0.001*	
	12 months	0.35 (0.64)	0.61 (0.97)	0.217	0.33 (0.64)	0.61 (0.97)	0.171
	Improvement from baseline	−2.35 (1.75)	−2.73 (2.12)	0.140	−2.38 (1.76)	−2.73 (2.12)	0.170
	*P*-value	< 0.001*	< 0.001*		< 0.001*	< 0.001*	

FAS, full analysis set; PPS, per protocol set; LKS, Lysholm Knee Score; TAS, Tegner Activity Score; SD, standard deviation

* *P* < 0.05

A post-hoc subgroup analysis was conducted for patients with isolated meniscal tears (*n* = 18; intervention group: *n* = 8; control group: *n* = 10) to assess potential confounding by concomitant ACL reconstruction. At 6 months, the mean (SD) LKS improvement was 28.63 (17.62) in the intervention group versus 29.90 (16.87) in the control group (*P* = 0.156). Due to the small subgroup size (*n* = 18), these results are exploratory and lack statistical power, limiting their persuasiveness. (Table 6).

**Table 6:** Clinical outcomes on secondary endpoints for the two groups (isolated tears).

Outcomes mean (SD)	Interval	Intervention group (*n* = 8)	Control group (*n* = 10)	*P*-Value (groups)
LKS	Baseline	62.88 (16.06	59.60 (16.15	0.674
	3 months	80.13 (14.22)	75.60 (13.65)	0.502
	Improvement from baseline	17.25 (25.98)	16.00 (18.80)	0.907
	*P*-value (pre–post)	< 0.102	< 0.025*	
	6 months	91.50 (8.96)	89.50 (9.52)	0.656
	Improvement from baseline	28.63 (17.62)	29.90 (16.87)	0.878
	*P*-value (pre–post)	< 0.001*	< 0.001*	
	12 months	96.00 (5.81)	93.40 (7.17)	0.291
	Improvement from baseline	33.13 ± 18.83	33.80 (18.24	0.940
	*P*-value	<0.001*	0.002*	
TAS	Baseline	4.25 (0.71)	4.40 (0.97)	0.719
	3 months	2.50 (1.20)	2.80 (0.79	0.531
	Improvement from baseline	−1.75 (1.28)	−1.60 (1.17)	0.799
	*P*-value	0.006*	0.002*	
	6 months	3.63 (1.19	3.70 (0.67)	0.868
	Improvement from baseline	−0.63 (1.51	−0.70 (1.34)	0.912
	*P*-value	0.279	0.132	
	12 months	4.13 (1.13)	4.40 (1.71)	0.701
	Improvement from baseline	−0.13 (1.64)	0.00 (1.94)	0.887
	*P*-value	0.836	1.000	
VAS	Baseline	2.88 (1.55)	3.00 (1.49)	0.864
	3 months	1.00 (0.93)	1.70 (1.34)	0.227
	Improvement from baseline	−1.88 (1.81)	−1.30 (2.00)	0.537
	*P*-value	0.022*	0.07	
	6 months	0.50 (0.76)	1.00 (0.82)	0.201
	Improvement from baseline	−2.38 (1.60)	−2.00 (1.76)	0.647
	*P*-value	0.004*	0.006*	
	12 months	0.25 (0.46)	1.10 (1.45)	0.108
	Improvement from baseline	−2.63 (1.51)	−1.90 (2.42)	0.472
	*P*-value	0.002*	0.035*	

* *P* < 0.05

#### MRI at 6 and 12 months postoperatively

At 6 months postoperatively, among patients with unilateral tears, no retear cases were observed in the intervention group, while three cases (9.68%) showed retear in the control group. Neither group showed implant destruction or breakage, and no displacement or loosening in the intervention group. However, one case (3.23%) in the control group showed signs of displacement from MRI. For patients with bilateral tears at 6 months postoperatively, one case in each group showed implant displacement or loosening. No retear cases were observed in either group. Based on FAS or PPS analysis, all patients showed synovitis, with most cases reduced compared with preoperative levels. Effusions and bone marrow edema were comparable between the groups and also reduced compared with preoperative conditions. Although some individual indices differed between the groups, these differences were not statistically significant except for synovitis compared with preoperative conditions in patients with bilateral tears (*P* = 0.014; Table 7)

**Table 7:** MRI evaluations at postoperative 12 months for the two groups.

Tear type	Parameter	Outcome	Intervention group, No. (%)	Control group, No. (%)	*P*-Value
Unilateral tear		Total (Missing)	33 (0)	30 (0)	-
	Signal classification	Level 0	0 (0.00%)	0 (0.00%)	1.000
		Class I	4 (12.12%)	4 (13.33%)	
		Class II	4 (12.12%)	4 (13.33%)	
		Class III	25 (75.76%)	22 (73.33%)	
	Retear or not	Yes	1 (3.03%)	0 (0.00%)	1.000
		No	32 (96.97%)	30 (100.00%)	
	Implant destruction or breakage	Yes	0 (0.00%)	0 (0.00%)	-
		No	33 (100.00%)	29 (100.00%)	
	Implant displacement/dislocation	Yes	0 (0.00%)	0 (0.00%)	-
		No	33 (100.00%)	29 (100.00%)	
	Perimeniscal synovitis	Yes	33 (100.00%)	28 (93.33%)	0.223
		No	0 (0.00%)	2 (6.67%)	
	Synovitis compared to baseline	Total (Missing)	31 (2)	28 (0)	
		Reduced	27 (87.10%)	26 (92.86%)	1.000
		Aggravated	1 (3.23%)	0 (0.00%)	
		Unchanged	3 (9.68%)	2 (7.14%)	
	Perimeniscal effusion	Total (Missing)	32 (1)	30 (0)	
		Yes	28 (87.50%)	22 (73.33%)	0.158
		No	4 (12.50%)	8 (26.67%)	
	Perimeniscal effusion compared to baseline	Total (Missing)	28 (0)	22 (0)	
		Reduced	5 (17.86%)	4 (18.18%)	1.000
		Aggravated	2 (7.14%)	1 (4.55%)	
		Unchanged	21 (75.00%)	17 (77.27%)	
	Perimeniscal bone marrow edema	Total (Missing)	33 (0)	30 (0)	
		Yes	3 (9.09%)	2 (6.67%)	1.000
		No	30 (90.91%)	28 (93.33%)	
	Perimeniscal bone marrow edema compared to baseline	Total (Missing)	3 (0)	2 (0)	
		Reduced	2 (66.67%)	2 (100.00%)	1.000
		Aggravated	1 (33.33%)	0 (0.00%)	
	Unchanged	0 (0.00%)	0 (0.00%)	
Bilateral tears		Total (Missing)	5 (0)	5 (0)	-
	Retear or not	Yes	0 (0.00%)	0 (0.00%)	-
		No	0 (0.00%)	0 (0.00%)	
	Implant destruction or breakage	Yes	0 (0.00%)	0 (0.00%)	-
		No	0 (0.00%)	0 (0.00%)	
	Implant displacement or dislocation	Yes	0 (0.00%)	0 (0.00%)	-
		No	0 (0.00%)	0 (0.00%)	
	Perimeniscal synovitis	Yes	5 (100.00%)	5 (100.00%)	1.000
		No	0 (0.00%)	0 (0.00%)	
	Perimeniscal effusion	Yes	5 (100.00%)	4 (80.00%)	0.048*
		No	0 (0.00%)	1 (20.00%)	
	Perimeniscal bone marrow edema	Yes	5 (100.00%)	1 (20.00%)	-
		No	0 (0.00%)	4 (80.00%)	

* *P* < 0.05

At 12 months postoperatively, among patients with unilateral tears, one case (3.03%) showed retear in the intervention group, with no retear cases in the control group. No implant destruction, fracture, displacement or loosening cases occurred in all patients. No significant differences were found in synovitis, effusions and bone marrow edema (Table 7).

### Safety Outcomes

Safety analysis showed no significant difference in the incidence of surgery-related AEs between the groups (17.02% vs 17.39%, *P* = 1.000). Device-related AEs occurred in 8.51% (4/47) of the intervention group versus 2.17% (1/46) in the control group. These included intraoperative device dysfunctions, such as suture slippage or deployment failures, which were managed during the procedure by using additional implants without necessitating surgical revision or affecting postoperative recovery. No serious device-related AEs were observed, and MRI evaluations confirmed low rates of retear or implant issues at follow-up (Table 8).

**Table 8:** Description of adverse events (AEs).

	Intervention group (*N* = 47)			Control group (*N* = 46)			*P*-Value
Item	Frequency	Patient No.	Incidence (%)	Frequency	Patient No.	Incidence (%)	
Surgery-related AEs	11	8	17.02%	9	8	17.39%	1.000
Device-related AEs	4	4	8.51%	1	1	2.17%	0.361
SAEs	0	0	0.00%	0	0	0.00%	-
Surgery-related SAEs	0	0	0.00%	0	0	0.00%	-
Device-related SAEs	0	0	0.00%	0	0	0.00%	-

AE, adverse event; SAE, serious adverse event.

## Discussion

This RCT demonstrated that the clinical safety and efficacy of the all-inside, all-suture meniscal repair device is not inferior to the widely used all-inside meniscal repair system in repair of vertical longitudinal full-thickness meniscus tears. Beyond validation, the device’s innovative all-suture, knotless mechanism provides clinical advantages, including reduced soft tissue stimulation and superior early Lysholm improvements (*P* = 0.015 at 6 months), positioning it as a refined alternative to established rigid-anchor systems.

Meniscal repair techniques have evolved from the traditional inside-out suture to outside-in and all-inside sutures, each with unique advantages and clinical manifestations. The inside-out suture allows for vertical sutures and is currently the most preferred technique. This technique requires an additional incision and an assistant to tie knots on the capsule, making it technically demanding and time-consuming. Previous studies have reported an association with higher rates of saphenous nerve injury (medial meniscal repairs) and common peroneal nerve injury (lateral meniscal repairs)^[32,33]^. The outside-in technique was first described by Warren *et al* to decrease the risk of peroneal nerve injury, which is suitable for repairing the anterior and midbody of the meniscus^[34]^. As a systematic review summarized, this technique effectively improves the quality of life and activity levels of patients with acute meniscal tears^[35]^. However, it still has disadvantages, such as complex intra-articular maneuvers and occasional need for extra devices^[36]^.

All-inside technique and repair sutures have been widely used in recent years. The all-inside suture was designed for minimally invasive closure of torn menisci using a built-in suture and specialized instruments. Earlier generation devices, such as PEEK and bioabsorbable anchors, demonstrated comparable healing rates to traditional suture techniques. However, these devices raised concerns regarding implant-induced synovitis, cyst formation, cartilage damage, and strength level^[37,38]^. Newer all-inside anchors have subsequently been developed utilizing soft materials. They are deployed posterior to the capsule and expand upon tensioning the repair^[39,40]^. The all-inside suture technique significantly reduces the risk of nerve injury compared with conventional suture techniques. Previous analysis showed that the risk of nerve injury was 85% lower with the all-inside suture than with the conventional suture. Despite the low complication rate, a few patients still experienced SAEs or device-related adverse reactions^[38]^.

The all-suture, all inside and knotless meniscal repair system represents one of the latest all-inside devices. It builds upon the foundational principals of the soft anchor implant and ZipLoop™ technology (Zimmer Biomet, Warsaw, In). It is a second-generation knotless device that utilizes a 1.6-mm needle inserter to deploy two soft polyester suture anchors linked by a 2–0 ultra-high molecular weight polyethylene (UHMWPE) adjustable locking suture. The locking suture is tensioned via a two-step process that independently tensions the two strands tightly and equally. In terms of biomechanical performance, an evaluation comparing JuggerStitch and Ultra Fast-Fix in a human cadaveric model showed there no significant differences in cyclic or static loading^[41]^. Massey *et al* further compared all-suture devices in cadaveric models, confirming superior failure loads for all-suture repairs in longitudinal tears^[42]^. The weaker and more variable cadaveric tissue led to elongation and catastrophic failures at the clamp-tissue interface and the suture-tissue interfaces for both devices. However, testing in porcine tissue reduced tissue failure incidence for both devices compared with cadaver tissue, revealing it’s superior strength during static loading (*P* = 0.004). In a study by Lawrie *et al*^[43]^, the deployment failure of all-inside meniscal repair system occurred in 17.43% of cases, possibly due to poor meniscus quality or design issues, which was in line with the intraoperative deployment failure rate in this study (15.85%). In our study, intraoperative device success rates were comparable between groups (*P* = 0.180), with failures limited to minor suture slippage during tensioning, resolved without sequelae. These align with reported all-inside repair incidences which influenced by factors such as meniscal tissue quality, tear location, and insertion angle. In addition, surgeons in our study determined that these failures were also significantly related to the learning curve.^[44–46]^ Since these pullout anchors and connected sutures were removed and replaced with new implants without harming the patient, clinical efficiency of meniscus repair was not affected. The lower immediate implant success rate in the intervention group (84.15%) may relate to the learning curve for the all-suture device, consistent with deployment challenges reported in similar systems. However, Massey *et al* confirmed its biomechanical superiority in failure load for longitudinal tears, suggesting that with familiarity, outcomes remain favorable^[42]^. Villarreal-Espinosa *et al* also reported similar complication rates in all-inside repairs, supporting that learning curves contribute to initial failures but do not compromise long-term efficacy^[32]^. To standardize judgments, all surgeons followed a predefined protocol for assessing immediate success and received module training to practice the use of devices, mitigating variability in learning curves across the multi-center design.

In terms of the efficacy of the all-inside, all-suture meniscal repair device, our study showed higher LKS score of 96.02 at 12 months, suggesting that as an improved version of meniscal repair device, the enhanced inserter tip design enhances ease of use and minimizes meniscal tissue cutting during insertion. The study also presented significant improvements in LKS, TAS, and VAS for pain up to 12 months in both groups, which are also comparable to the results demonstrated by the widely used all-inside meniscal repair system^[30]^. Moreover, the intervention group in this study showed significantly higher LKS improvements at 6 months, indicating superior early stage knee function recovery. For the safety, MRI evaluations showed that the all-inside, all-suture meniscal repair device had a low incidence of postoperative meniscus retear, destruction, breakage and displacement of implant as the controlled device. The safety and stability of the implant had also been demonstrated by significant reductions in peri-meniscal synovitis, effusion, bone marrow edema, and smooth recovery process at 12 months postoperatively.

The presence or absence of other injuries and concomitant structural knee injuries can significantly influence the success rate of meniscus repair. Hagino *et al* found that meniscus tears occurred in a rate of 79.2% patients with ACL injuries, including 72.7% in the acute group and 84.8% in the chronic group^[47]^. Published data revealed that meniscal healing success rates reach 75–92% with concomitant ACL reconstruction, compared with 63% without ACL reconstruction^[48]^. ACL reconstruction leads to the formation of articular and fibrin clot, which provides intra-articular growth factors and a repair scaffold conducive to meniscus healing, thereby increasing the likelihood of successful repair. Several researchers have shown that meniscus repair with concomitant ACL reconstruction could achieves superior clinical outcomes and reduces the risk of meniscus repair failure, recommending this combined approach^[48,49]^. The high rate of concomitant ACL reconstruction (78.49%) in our cohort may have influenced outcomes, as such procedures are associated with improved meniscal healing and lower failure rates compared to isolated repairs^[50]^. To mitigate this potential bias, a subgroup analysis of isolated tears showed trends consistent with the overall results, though non-significant due to small sample size. This supports the robustness of our findings but highlights the need for dedicated studies on isolated meniscal repairs.

In summary, this rigorously designed multicenter RCT study with level 1 evidence validated the efficacy and safety of the all-inside, all-suture meniscal repair device across multiple parameters and visit intervals, including physical examinations, patient-reported outcomes, radiographic evaluations and safety performance throughout the study period. Current results demonstrate comparable efficiency and safety to other all-inside meniscal repair devices, with the advantages of soft materials and enhanced mechanical properties.

The limitations of our study need to be acknowledged. First, the follow-up period was limited to postoperative 12 months, which does not offer insights into long-term outcomes, such as re-tear rates, osteoarthritis progression or complications on implanted anchors. While this duration is sufficient for evaluating functional recovery, as evidenced by stabilization of PROMs and returning to activity levels typically within 6–12 months in similar studies^[12]^, long-term follow-up is also necessary to assess repair durability and potential late complications. An extended follow-up to 24–36 months is ongoing. Second, the study included only patients with vertical longitudinal full-thickness meniscus tears in vascular zones (red–red/red–white), as per the device’s regulatory indications at initiation. This tear type is common in young, active individuals (50–90% of pediatric/adolescent tears) and has high healing potential due to vascularity^[8,51]^, making it ideal for device evaluation but limiting generalizability to other patterns like degenerative or avascular tears, which predominate in older populations and may yield poorer outcomes. Massey *et al* similarly tested longitudinal tears in cadaveric models, reinforcing biomechanical efficacy for this pattern but highlighting the need for studies on broader tear types^[42]^. Further research is needed to evaluate its performance in broader tear patterns. Third, while MRI was used to assess meniscus repair status at 6 and 12 months postoperatively, MRI findings alone may not fully reflect clinical success. Retears detected on MRI do not necessarily indicate failure in terms of functional recovery and pain relief, as scar tissue signals can persist for years or indefinitely, potentially mimicking retears without symptomatic correlation. In this study, one patient in the intervention group showed a retear on MRI at 12 months but experienced no functional limitations, increased pain, or need for secondary arthroscopy to confirm the retear status. This aligns with prior research showing a disconnect between post-repair MRI signals and clinical outcomes, highlighting that MRI should be interpreted in the context of symptoms rather than as a standalone measure of repair failure^[12]^.

## Conclusion

This study demonstrated that the efficacy and safety of the all-inside, all-suture meniscal repair device are comparable to that of the controlled all-inside meniscal repair device for arthroscopic meniscal repair on vertical longitudinal full-thickness meniscus tears in the short-term follow-up. Additionally, the all-inside, all-suture meniscal repair device significantly promoted early knee function recovery. Our findings suggest that the all-inside, all-suture meniscal repair device is a viable and effective alternative for arthroscopic meniscal tear repair.

## Data Availability

The datasets generated and/or analyzed during the current study are not publicly available due to privacy considerations and institutional data policies. However, individual participant data (after de-identification) may be made available upon reasonable request to the corresponding author, subject to approval by the ethics committees of the participating institutions and compliance with data protection regulations.

## Research registration unique identifying number (UIN)

ClinicalTrials.gov, ID: NCT06899659.
